# Study protocol of the CHOiCE trial: a three-armed, randomized, controlled trial of home-based HPV self-sampling for non-participants in an organized cervical cancer screening program

**DOI:** 10.1186/s12885-016-2859-z

**Published:** 2016-11-03

**Authors:** Mette Tranberg, Bodil Hammer Bech, Jan Blaakær, Jørgen Skov Jensen, Hans Svanholm, Berit Andersen

**Affiliations:** 1 Department of Public Health Programmes, Randers Regional Hospital, Skovlyvej 15, 8930 Randers, NØ Denmark; 2 Department of Public Health, Section for Epidemiology, Aarhus University, Bartholins Allé 2, 8000 Aarhus C, Denmark; 3 Department of Obstetrics and Gynecology, Aarhus University Hospital, Palle Juul-Jensens Boulevard 99, 8200 Aarhus N, Denmark; 4 Statens Serum Institut, Artillerivej 5, 2300 Copenhagen S, Denmark; 5 Department of Pathology, Randers Regional Hospital, Østervangsvej 48, 8930 Randers, NØ Denmark; 6 Department of Clinical Medicine, Aarhus University, Palle Juul-Jensens Boulevard 82, 8200 Aarhus N, Denmark

**Keywords:** Self-sampling, Cervical cancer screening, Screening participation, Human papillomavirus testing

## Abstract

**Background:**

The effectiveness of cervical cancer screening programs is challenged by suboptimal participation and coverage. Offering cervico-vaginal self-sampling for human papillomavirus testing (HPV self-sampling) to non-participants can increase screening participation. However, the effect varies substantially among studies, especially depending on the approach used to offer HPV self-sampling. The present trial evaluates the effect on participation in an organized screening program of a HPV self-sampling kit mailed directly to the home of the woman or mailed to the woman’s home on demand only, compared with the standard second reminder for regular screening.

**Methods/design:**

The CHOiCE trial is a parallel, randomized, controlled, open-label trial. It will include 9327 women aged 30–64 years who are living in the Central Denmark Region and who have not participated in cervical cancer screening after an invitation and one reminder. The women will be equally randomized into three arms: 1) Directly mailed a second reminder including a HPV self-sampling kit; 2) Mailed a second reminder offering a HPV self-sampling kit, to be ordered by e-mail, text message, phone, or through a webpage; and 3) Mailed a second reminder for a practitioner-collected sample (control group). The primary outcome will be the proportion of women in the intervention groups who participate by returning their HPV self-sampling kit or have a practitioner-collected sample compared with the proportion of women who have a practitioner-collected sample in the control group at 90 and 180 days after mail out of the second reminders. Per-protocol and intention-to-treat analyses will be performed. The secondary outcome will be the proportion of women with a positive HPV self-collected sample who attend follow-up testing at 30, 60, or 90 days after mail out of the results.

**Discussion:**

The CHOiCE trial will provide strong and important evidence allowing us to determine if and how HPV self-sampling can be used to increase participation in cervical cancer screening. This trial therefore has the potential to improve prevention and reduce the number of deaths caused by cervical cancer.

**Trial registration:**

Current Controlled Trials NCT02680262. Registered 10 February 2016.

## Background

Organized screening programs have reduced cervical cancer incidence and mortality in many western countries [1–3]. Yet, the effectiveness of such programs is challenged by suboptimal participation and coverage [4, 5], and more than half of all invasive cervical cancers are diagnosed among women who are under- or unscreened [6–8]. It is therefore crucial to identify ways to improve screening participation, e.g. by removing existing barriers to regular screening.

In the Danish organized screening program, the overall participation rate, defined as having a screening test within 365 days after an invitation, is currently 65 % [9]. This percentage has been decreasing slightly in recent years [9]. Earlier studies show sociodemographic inequalities in screening participation [10, 11]. Thus, a Danish study identify several barriers to participation, including lack of knowledge about screening and cervical cancer, discomfort during pelvic examinations, fear of cancer, practicalities in private life, and in access to testing facilities [12]. Some of these barriers may be overcome if self-sampling at home is an option which, however, is not currently the case in the Danish screening program.

Recent research advocates the use of high-risk human papillomavirus (hrHPV) testing over cytology because it is more sensitive in detecting cervical intraepithelial neoplasia of grade 2 or worse (CIN2+) and provides better protection against cervical cancer [13, 14]. Furthermore, hrHPV testing enables women to self-sample cervico-vaginal material; self-collected samples have shown sensitivity for detection of CIN2+ that is comparable to that of clinician-collected samples if validated Polymerase Chain Reaction (PCR)-tests are used [15, 16]. Self-sampling also appears to improve screening participation. Hence, a meta-analysis showed that mailing women a test-kit for self-sampling at home, including pre-stamped envelopes for mailing of the sample to a laboratory for HPV testing, increased screening participation compared with women receiving standard invitation for regular screening [17]. The participation rate for women offered self-sampling varies widely among trials, ranging from 10 % [18] to 39 % [19] with a pooled 12.6 % absolute increase in participation compared with standard invitation when self-sampling kits were mailed directly to all women [17].

Three other trials [20–22] used an opt-in approach for offering self-sampling, i.e. women were mailed an invitation to order a kit by phone [21], or by mail [20], or to pick it up at a pharmacy [22]. One of these trials [20] showed a 12.3 % increase in participation among long-term non-participants, but the two other trials [21, 22] showed no positive effect on participation. Moreover, in the pooled analysis, these trials showed only an insignificant 0.2 % participation increase compared with women receiving standard invitations [17]. Thus, more studies are needed to explore the effect and acceptability associated with other more timely and modern opt-in approaches for offering self-sampling (websites, e-mails, and text messages). To our knowledge, no previous studies have evaluated the effect on participation of offering self-sampling, either directly mailed or using timely opt-in procedures as compared with a standard second reminder.

Therefore, in the efforts to reduce barriers to cervical cancer screening and to increase participation, we will conduct a randomized, controlled effectiveness trial to evaluate the effect of two different approaches for offering HPV self-sampling to women who did not participate in the cervical cancer screening program despite an invitation and one reminder.

## Methods

### Trial design

**Fig. 1:**
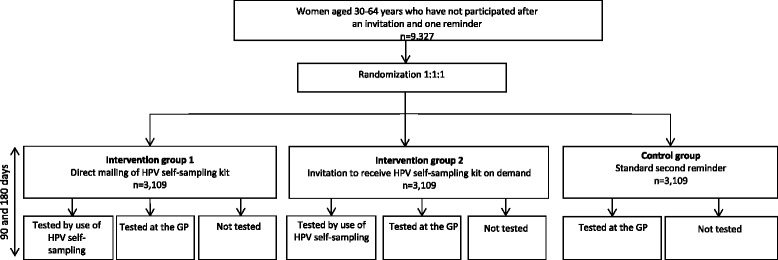
Randomized controlled trial design overview. GP: general practitioner

CHOiCE (Cervical HOme-based CancEr screening) is a parallel, randomized, controlled, open-label trial nested into a population-based, organized cervical cancer screening program conducted in the Central Denmark Region. Women who have not participated in cervical cancer screening after an invitation and one reminder will be randomly allocated to one of the following three arms (Fig. 1):

1. mailing of a modified second reminder including the self-sampling kit (intervention group 1)
2. mailing of a modified second reminder informing the women that they can order the kit either by e-mail, text message, phone or through a study webpage (www.hjemme-us.rm.dk) (intervention group 2)
3. mailing of a standard second reminder (control group)

The modified second reminder informs of the opportunity to collect a self-sample if wanted, but also about the opportunity to have a cervical cytology specimen taken at a general practitioner (GP) (usual procedure). The standard second reminder informs the women about the current test opportunity, but contains no information about self-sampling.

### Study setting

Denmark has a total of 5.6 million inhabitants, with 1.5 million women in the target population for cervical cancer screening [9, 23]. Organized cervical cancer screening was introduced in the 1960s in some Danish counties and non-systematically implemented in the rest of the country until nationwide coverage was achieved in the late 1990s [24, 25]. The policy and organization of cervical cancer screening are defined nationally, but the responsibility for running the screening program lies with the regions [26, 27]. Denmark is divided into five regions one of which is the Central Denmark Region (23 % of the Danish population) [23, 26].

Since 2007, women aged 23–49 years have been invited for cervical cancer screening every third year, while women aged 50–64 years are invited every fifth year [26, 27]. Shortly before 3 or 5 years have passed since a woman’s last registered cervical cytology result, she is sent an invitation advising her to book an appointment with a GP for a pelvic examination at which a liquid-based cytology specimen is collected [27]. If the responsible Department of Pathology does not receive a cervical cytology specimen for analysis, up to two reminders will be sent 3 and 6 months after the initial invitation [27]. Women who do not respond to invitations or reminders will receive a new invitation in the next screening round, unless she has declined participation. For women aged 23–59 years, the primary screening test is liquid-based cytology; while an hrHPV-DNA check-out test is recommended for women aged 60–64 years [26, 27]. In the Danish Cervical Cancer Screening Program, all testing is free of charge [26, 27].

Routinely, all cervical cytology results, HPV test results, and histological diagnoses from cervical biopsies as well all other pathology specimens are registered in the national Danish Pathology Data Bank (DPDB) under the woman’s unique Civil Personal Registration (CPR) number [28–30]. The DPDB also keeps track of which women are due to receive invitations and reminders to participate in screening.

In the Central Denmark Region, all samples in the Cervical Cancer Screening Program are analyzed by the Department of Pathology, Randers Regional Hospital. Invitations and reminders are routinely handled by the Department of Public Health Programs, Randers Regional Hospital.

### Participation and randomization

Participants are restricted to women aged 30 to 64 years living in the Central Denmark Region who have not participated in cervical cancer screening after an invitation and one reminder. Women who are younger than 30 years are not included due to the low specificity of HPV DNA tests in younger women [14].

Participants will be identified on a weekly basis in the nationwide DPDB [28, 29]. The eligible women’s unique 10-digit CPR number [30], including birthdate will be extracted as this allows us to follow, e.g. the women in Danish health registers. A CPR number is assigned to every Danish citizen upon birth [30]. Data will be extracted in Excel format and transferred to the REDCap system for storage and automated randomization [31]. Participants will be assigned randomly to the three arms of the trial in a 1:1:1 ratio as per a computer-generated randomization schedule following simple randomization procedures. The CPR number is used for randomization. The nature of the intervention and allocation ratio precludes the masking of the participants and study staff.

### Interventions

Women in intervention group 1 will be mailed a modified second reminder, a leaflet entitled *Facts*, *benefits, and drawbacks about HPV self-sampling* and a self-sampling kit. The leaflet provides information about HPV and cervical cancer including benefits and drawbacks of HPV self-sampling compared with regular screening. The kit includes a brush device (Evelyn Brush, Rovers Medical Devices B.V, Oss, Netherlands), which should be used to collect a cervico-vaginal sample for subsequent hrHPV testing [32], written and drawn instructions on how to obtain and mail the sample, and a pre-stamped return envelope addressed to the Department of Pathology, Randers Regional Hospital, where the hrHPV testing will be performed. The instructions show how the woman should label the brush device with an attached laboratory specimen barcode. The woman is urged to mail the return envelope on the day the sample is taken. Women in intervention group 2 receive the same material as those in intervention group 1, except for the kit, which will be mailed to the women only on demand. Additionally, the leaflet for this group contains information on how to order the kit (by e-mail, text message, phone, or through a study webpage (www.hjemme-us.rm.dk). Women in both intervention groups and in the control group receive the information that they can contact their GP should they wish to have a cervical cytology specimen taken.

### Analysis of samples

All samples will be handled, processed and analyzed at the Department of Pathology, Randers Regional Hospital.

The self-collected samples will be handled using the Cobas® 4800 HPV DNA test (Roche Diagnostics GmBH, Switzerland) according to the manufacturer’s protocols. The test identifies HPV16, HPV18 and 12 other hrHPV types (31, 33, 35, 39, 45, 51, 52, 56, 58, 59, 66 and 68) in a single pool. Results are either 1) hrHPV-negative, 2) hrHPV-positive (HPV16, HPV18 and/or other hrHPV types), or 3) unsatisfactory. An unsatisfactory result includes specimens with a negative β-globin result, a sample damaged in transit, incorrect labelling, or insufficient material for analysis. The hrHPV test results of the self-collected samples will be registered in the DPDB.

Cervical cytology specimens obtained by GPs will be analyzed using the standard procedure used in the Central Denmark Region, i.e. microscopy as the primary procedure for control group women aged 23–59 years [27]. In case of detection of Atypical Squamous Cells of Undetermined Significance (ASC-US) among women aged ≥ 30 years, an HPV DNA analysis will be performed using Cobas 4800 [9, 27]. For women aged 60–64 years, the primary analysis of the cervical cytology is Cobas 4800 HPV DNA analysis, and microscopy will be used as triage in case of hrHPV-positive test results [9, 27]. When cervical cytology is made as follow-up on hrHPV-positive test results following self-sampling (see below), the specimens will be analyzed by microscopy.

### Follow up after self-sampling

Women tested by use of self-sampling receive a written test result by ordinary mail. Approximately 98 % of all residents in Denmark are listed with a GP [33]. The GP will also be informed of the testing and the test result. Women with an hrHPV-positive test results are recommended to visit their GP for a cervical cytology specimen within 30 days; and hereafter they will be handled as described in Danish routine guidelines [27]. Women with an hrHPV-negative test result will be referred back to the national screening program and recommended to participate in the next screening round. Women with an unsatisfactory sample will receive a second self-sampling kit and will be encouraged to repeat self-sampling at home or to visit a GP for a cervical cytology specimen.

### Sample size

The sample size was determined by the primary objective (comparison of participation in the intervention and control groups, respectively) and women will be allocated in equal numbers to the three randomization groups. Our assumption about participation in the control group (women who have a cervical cytology specimen within 90 days after receiving the standard second reminder) is 28.7 % [34]. A power calculation (considering a 2.5 % significance level and 80 % power) based on finding an expected difference of 3.6 % [35] in participation between the intervention groups and the control group shows that the trial will achieve a statistical power of 80 % if 3109 women are included in each group (a total of 9327 women).

### Data sources and statistical analysis

The DPDB will be used to retrieve data on the CPR numbers of the study population; participation (yes/no); if participation was by self-sampling or by visiting a GP; the hrHPV test results of the self-collected samples, results of cervical cytology specimens, results of cervical biopsies and whether appropriate follow-up was conducted (only for self-sampling women). Furthermore, data on the women’s previous screening history will be obtained from the DPDB. From Statistics Denmark, information on sociodemographic status will be obtained using the women’s CPR number. An overview of the used data sources and information is seen in Table 1.

The characteristics of the women in the intervention groups and the control group will accordingly be presented using descriptive statistics (mean, standard deviation, numbers, or proportions) on sociodemographic factors (e.g. age, marital status, education level, ethnicity, income, living in rural or urban area, and occupation) and previous screening history in order to determine if the randomization was adequately balanced.

Participation will be defined as a submitted self-collected sample or having a cervical cytology specimen within 90 and 180 days after the mailing of second reminders. The proportion of women participating in each group will be calculated, as will the absolute difference in the participation proportion between the control and intervention groups and the corresponding 95 % confidence intervals (CIs). The relative risks and 95 % CIs of having a sample in the intervention groups compared with the control group will be estimated. Per-protocol and intention-to-treat analyses will be performed. The latter include data on women who were invited to self-sample, but instead attended regular screening. Participation will also be reported by age and screening history. The prevalence of hrHPV cases and histologically confirmed CIN lesions in the interventions groups will be reported. Estimates and 95 % CIs of the proportion of women with a hrHPV sample who have appropriate follow-up will also be calculated. Appropriate follow-up will be defined as having a cervical cytology specimen taken at 30, 60, or 90 days after mailing of the test result.

**Table 1:** Overview of data sources and information

Data sources	Information
Danish Pathology Data Bank (DPDB)	Participation (yes/no)
	Participation by self-sampling or visiting a GP
	HrHPV test results of self-collected samples
	Dysplasia and/or hrHPV test results of cervical cytology and histology specimens obtained in the whole country
	Screening history
	Age at date of second reminder
Statistic Denmark	Marital status
	Living in rural or urban area
	Education level
	Ethnicity (country of birth)
	Occupation
	Income

All data are registered and collected by use of the unique CPR number which includes the participant’s date of birth

*CPR* Civil Personal Registration, *GP* General Practitioner

### Participant timeline

The study will continue until a total of 9327 women have been invited. The expected study duration, including the follow-up period, is 12 months. Kits and reminders will be sent out progressively over an estimated 4-month-period starting in March 2016.

### Ethical considerations

The study was approved by the Danish Data Protection Agency (j. no.: 1-16-02-495-15) and by Danish Health Authorities (j. no.: 3-3013-1407/1). The study has been presented to The Central Denmark Region Committees on Health Research Ethics. The Committeesdecided that, according to the Danish Act on Research Ethics Review of Health Research Projects (Act number 593 of 14 July 2011),this study should not be notified to the Committees (j. no.: 1-10-72-259-15).

Included women receive written information about the self-sampling procedure and the drawbacks and benefits of self-sampling versus regular screening. Likewise, the information includes a passage that clearly explains that if hrHPV is detected, the woman will be referred for subsequent follow-up testing. Any woman who returns the self-collected sample hereby expresses her consent to the analysis of the sample and to receiving any test results and follow-up recommendations by mail. The women are also informed that their GP will be informed of their test result.

## Discussion

The Danish Cervical Cancer Screening Program is challenged by a suboptimal participation rate [9]. Nearly half (45 %) of all newly diagnosed cervical cancers in Denmark are found among under-screened women [36]. Numerous other countries are facing a similar situation [6, 8]. Strategies to improve participation are important priorities for the Cervical Cancer Screening Program, and new strategies are needed to target women who have not participated in cervical cancer screening despite invitations for regular screening [27]. HPV self-sampling is a valid screening tool that has the potential to overcome known barriers to regular screening as evidenced by trials [18–22, 35, 37–44] showing that self-sampling can improve screening participation, although the effect varied substantially between countries. In addition, high compliance to follow-up recommendations among self-sample hrHPV-positive women is necessary to achieve the wanted benefit of the intervention and a meta-analysis by Verdoodt et al. [17] found that appropriate follow-up was achieved only in 82 % of women with a hrHPV-positive test result. Evidence from Denmark is therefore necessary to inform policy makers before introducing self-sampling.

The planned study gains validity from the fact that all Danish women have a unique CPR number and that all activities in the Danish healthcare system, including those related to cervical cancer screening, are registered using this number. This enables linkage to e.g. information on previous screening history which allows us to determine the capacity of self-sampling to recruit under- or unscreened women. The DPDB is a nationwide database that holds detailed, highly valid records on all pathology specimens, including cervical cytologies and HPV tests of provider-collected and self-collected samples from all Danish pathology departments [28, 29]. Another strength of the study is that the self-sampling procedure is embedded directly into a population-based, well-run organized screening program. Women accept to have their sample analyzed only by submitting it to the pathology department. Thus, the routines of this study can be transferred directly to daily routines with results that are expected to be similar to those of the present study. As the Central Denmark Region comprises a mix of urban and rural areas, we will also be able to disclose whether there are true urban-rural differences in the effect of self-sampling as suggested in an Italian trial [21]. This may, in turn, afford us with better opportunities for transferring the results to other regions. Furthermore, we include a wider variety of more timely opt-in approaches than earlier studies using opt-in approaches [20–22]. This may increase the effect of this procedure as compared with direct mailing of a test-kit. Overall, we therefore expect to be able to introduce an approach that is more cost-effective than earlier described approaches.

It is a limitation in our study that we use only one type of sample device, as differences in participation rates may hinge on the sample device chosen. However, two trials [45, 46] have compared the effects of a lavage and a brush self-sampling device on screening participation. These trials found a slightly higher participation with the brush device; i.e. the same brush device as used in our study. Another limitation is that the intervention is designed to target only hard-to-reach women by seeking to overcome barriers related to seeking a physician for a cervical cytology specimen. Other previously described barriers [12] are not targeted in this study; but such barriers will clearly need to be investigated in future research.

The obtained results will be compared with the results of other self-sampling trials. Of particular interest is to study trials performed in countries with organized screening programs. We will seek to explain any discrepancies in the results with reference to differences in the study designs, interventions, study populations, and settings.

As the trial is an effectiveness study nested into a routine screening program, the findings will provide strong and important evidence allowing us to determine if and how HPV self-sampling can be used to improve screening participation. The trial therefore has the potential to improve cervical cancer prevention and to reduce the number of deaths caused by cervical cancer.
